# Genomic Analysis of SXT/R391 Integrative Conjugative Elements From *Proteus mirabilis* Isolated in Brazil

**DOI:** 10.3389/fmicb.2020.571472

**Published:** 2020-10-20

**Authors:** Juliana L. Sato, Marina R. B. Fonseca, Louise T. Cerdeira, Maria C. B. Tognim, Thais C. M. Sincero, Mario C. Noronha do Amaral, Nilton Lincopan, Rodrigo S. Galhardo

**Affiliations:** ^1^Department of Microbiology, Institute of Biomedical Sciences, University of São Paulo, São Paulo, Brazil; ^2^Department of Infectious Diseases, Central Clinical School, Monash University, Melbourne, VIC, Australia; ^3^Department of Basic Health Sciences, State University of Maringá, Maringá, Brazil; ^4^Department of Clinical Analysis, Health Sciences Center, Federal University of Santa Catarina, Florianópolis, Brazil; ^5^Department of Clinical Analysis, University of São Paulo, São Paulo, Brazil

**Keywords:** ICE, SXT/R391, CMY-2, *Proteus mirabilis*, WGS, nanopore, resistance

## Abstract

Integrative conjugative elements (ICEs) are widespread in many bacterial species, often carrying antibiotic resistance determinants. In the present work, we screened a collection of *Proteus mirabilis* clinical isolates for the presence of type 1 SXT/R391 ICEs. Among the 76 isolates analyzed, 5 of them carry such elements. The complete sequences of these elements were obtained. One of the isolates carried the CMY-2 beta-lactamase gene in a transposon and is nearly identical to the element ICE*Pmi*Jpn1 previously described in Japan, and later shown to be present in other parts of the world, indicating global spread of this element. Nevertheless, the Brazilian isolate carrying ICE*Pmi*Jpn1 is not clonally related to the other lineages carrying the same element around the world. The other ICEs identified in this work do not carry known antibiotic resistance markers and are diverse in variable gene content and size, suggesting that these elements may be responsible for the acquisition of other advantageous traits by bacteria. Some sequences carried by these elements in Brazilian strains were not previously found in other SXT/R391 variants.

## Introduction

*Proteus mirabilis* is one of the most common causes of urinary tract infections (Armbruster and Mobley, 2012). This bacterium also has the potential to cause other diseases, including a number of nosocomial infections (O’Hara et al., 2000). *Proteus* is one of the genera of gram-negative bacteria in which ICEs (Integrative Conjugative Elements) of the SXT/R391 family are usually found, similarly to *Morganella*, *Providencia*, *Vibrio* and others (Wozniak et al., 2009). These mobile elements so far constitute the most commonly found family of conjugative transposons according to the ICEberg database (Liu et al., 2019). They have the ability to integrate in a specific locus in the chromosome. Type 1 SXT/R391 ICEs integrate into the 5’ portion of the *prfC* gene, while types 2, 3, and 4 integrate into the 3’ portion of the tRNA-Ser gene. (Hochhut and Waldor, 1999; Bioteau et al., 2018). Excision from the chromosome and conjugation are stimulated by DNA damaging conditions. A LexA-like repressor, SetR, is cleaved in a RecA-dependent manner (Beaber et al., 2004), leading to expression of the SetCD activators, which in turn increase the expression of genes related to transfer of the ICE (Poulin-Laprade et al., 2015). Once inside a new host, integrase (encoded by *int*) mediates site specific integration of the ICE in *prfC* in a way that a functional allele of this gene is maintained in the chromosome (Hochhut and Waldor, 1999). Notably, these elements are also able to mobilize other genomic islands *in trans* (Daccord et al., 2010), therefore playing an important role in microbial genomic plasticity.

The basic structure of these elements is more or less conserved among variants from different species, with a conserved core of genes involved in the basic functions of excision, conjugation and integration (Bioteau et al., 2018). Variable regions and insertion hotspots are points of new DNA insertion, in which genes related to antimicrobial and heavy metal resistance can be acquired. These elements are large and the insertion points often contain other types of genes unrelated to resistance, many of which with unknown function (Wozniak et al., 2009; Rodríguez-Blanco et al., 2016).

These elements have attracted a great deal of interest, due to their potential to spread resistance determinants. In *V. cholerae*, these elements were originally identified as conferring resistance to sulfamethoxazole, trimethoprim, streptomycin and chloramphenicol (Waldor et al., 1996). In *P. mirabilis*, many instances of occurrence of such elements have been reported. Interestingly, some *P. mirabilis* strains harbor ICE SXT/R391 variants which do not carry known antibiotic resistance determinants, including the type strain HI4320 (Pearson et al., 2008; Wozniak et al., 2009). This observation raises the question of which selective advantage would be conferred by such large elements not carrying resistance genes. Nevertheless, SXT/R391 elements also mediate resistance dissemination in *P. mirabilis*. Of particular interest and prevalence, the variant ICE*Pmi*Jpn1, originally identified in Japan, carries a CMY-2 beta-lactamase gene (Harada et al., 2010). This particular variant of SXT/R391 ICEs was subsequently identified in *P. mirabilis* isolates from human and animal origins in Spain, India, France, China, and Ireland (Mata et al., 2011; Aberkane et al., 2016; Lei et al., 2016; Mac Aogáin et al., 2016; Bie et al., 2017).

Other variants of this element were recently identified in different *P. mirabilis* strains, carrying resistance to fluoroquinolones, beta-lactams, fosfomycin, and a rRNA methyltransferase conferring resistance to oxazolidinones and lincosamides (Bie et al., 2017; Lei et al., 2018). In other *Proteus* species, SXT/R391 elements also carry resistance to multiple drugs (He et al., 2020). In order to gain further insight into the biology, evolution and role of these elements in antimicrobial resistance spread, we investigated the presence and genetic background of SXT/R391 ICEs in Brazilian *P. mirabilis* clinical isolates.

## Materials and Methods

### Screening of SXT/R391 Elements in *P. mirabilis* Isolates

Screening of SXT/R391 elements was performed using previously described primers to detect *int* (sxtintF: 5′ TCGATGATGGTCTCTAGCTG 3′ and sxtintR: 5′ TCAGTTAGCTGGCTCGATGC 3′) (Mata et al., 2011) and *rumA* (rumAF: 5′ TGGTGACCACACCAAATATCTC 3′ and rumAR: 5′ AAGCCAAGCGCCTTCGTATT 3′). *rumA* primers were designed in this study to amplify a conserved region in this gene, spanning nucleotides 47 to 418 of the *rumA* coding region in ICE*Pmi*USA1 from strain HI4320, used in this study as the prototypic SXT/R391 element from *P. mirabilis*. While *rumA* primers could detect genes from all types of SXT/R391 ICEs (1, 2, 3 and 4), primers for *int* are specific for type 1 elements, which are the focus of this study. Isolates were screened by colony PCR, using *rpoB* amplification as a positive control for the reaction by using primers rpoBF: 5′ GAATGTCAGATCCGTGGTGT 3′, and rpoBR: 5′ CCAACCGCAGAGAGATCATA 3′. A total of 76 *P. mirabilis* clinical isolates were investigated, which were collected between 2014 and 2015 from patients in hospitals located in the cities of São Paulo and Maringá, in Southeast and Southern Brazil, respectively (SisGen number A9D703D). PCR was performed as follows. 1 U of Taq DNA polymerase (Sinapse Inc., Brazil) was used in 25 μL reactions containing 20 ng of genomic DNA and 0.25 μM of each primer. The following cycling conditions were used: initial denaturation at 94°C for 5 min, 30 cycles of denaturation at 94°C for 1 min, annealing for 30 s and extension at 72°C (calculated for 1 min/kbp), followed by final extension at 72°C for 10 min. The annealing temperature of each reaction is: *int* - 51°C, *rumA* - 60°C, and *rpoB* - 54°C.

### Antimicrobial Susceptibility Assays

The antibiotic resistance profiles of ICE-harboring strains were determined by disk-diffusion method following guidelines from CLSIs (CLSI, 2016). The antibiotics tested were: amoxicillin-clavulanate (AMC) 20/10 μg, piperacillin-tazobactam (PPT) 100/10 μg, ampicillin (AMP) 10 μg, cephalothin (CFL) 30 μg, cefotaxime (CTX) 30 μg, ceftriaxone (CRO) 30 μg, cefepime (CPM) 30 μg, ceftazidime (CAZ) 30 μg, ertapenem (ETP) 10 μg, imipenem (IPM) 10 μg, meropenem (MER) 10 μg, nalidixic acid (NAL) 30 μg, ciprofloxacin (CIP) 5 μg, levofloxacin (LEV) 5 μg, norfloxacin (NOR) 10 μg, amikacin (AMI) 30 μg, gentamicin (GEN) 10 μg, tobramycin (TOB) 10 μg, and sulfamethoxazole-trimethoprim (SUT) 1.25/23.75 μg. The test was performed using Müeller-Hinton agar (MHA) plates and as a control we used the reference strain *Escherichia coli* ATCC 25922.

### Genome Sequencing and Assembly

For all strains, total genomic DNA was extracted from saturated cultures using the Wizard Genomic DNA Kit (Promega) and the genome sequencing was performed by MicrobesNG^1^ with Illumina HiSeq using a 250 bp paired end protocol. Only for strain PmBR607, the Oxford Nanopore Sequencing (ONT) was also performed. For this purpose, total DNA was extracted using the Purelink genomic DNA mini kit (Invitrogen), and libraries were prepared using the Rapid Barcoding Kit (SQK-RBK004), and sequencing was performed with FLO-MIN106D flowcell.

Illumina reads were filtered by quality and adapters were removed using trimomatic v.0.39^2^ with minimum quality threshold PHRED < 20 for passed reads. *de novo* assembly was achieved using SPAdes v. 3.11 (Bankevich et al., 2012). For ONT reads the quality filter was performed using guppy 2.1.3 and the hybrid assembly was obtained with Unicycler (Wick et al., 2017). The annotation was performed by NCBI Prokaryotic Genome Annotation Pipeline (PGAP).

For PmBR19, Sanger sequencing was also used to complete the gap between two contigs containing the ICE sequence. For this purpose, the gap region was amplified with primers designed based on PmBR19 genome sequence aligned to ICE*Pmi*Jpn1 (KT894734): tn10F (5′ TTCGTTGCTTGTGAGGTGAG 3′) and tn10R (5′ AAACAACGGCTGGAATGTGC 3′). Then the PCR product was purified with NucleoSpin Gel and PCR Clean-up Kit (Macherey-Nagel) and Sanger sequencing was performed with primers tn10F, tn10R and 3 more primers designed for the gap region based on ICE*Pmi*Jpn1 sequence Tn10.1 (5′ GCCACGAGTAAGTCTTCCCT 3′), Tn10.2 (5′ GTCAGCCTCTTATAGCCTAAAGT 3′) and Tn10.3 (5′ GCCACGCATTACTTGACTGT 3′). The consensus sequence obtained from this Sanger sequencing was used to obtain the full sequence of ICE*Pmi*Jpn1 from PmBR19. The genome assemblies were deposited in Genbank BioProject database under accession code PRJNA576511.

### Conjugation Assays

Filter mating assays were performed to test ICE*Pmi*Jpn1 (PmBR19) mobility. Overnight cultures of the donor strain PmBR19 (carrying ICE*Pmi*Jpn1 conferring resistance to Ampicillin) and recipient strain *E. coli* MG1655 Rif^*R*^, were harvested by centrifugation and mixed at 1:1 ratio. The mixture was plated onto a sterile 0.22 μm pore size membrane filter placed on a non-selective MacConkey agar, and incubated overnight at 37°C. Then the filter was washed with LB broth and the non-diluted mixture was plated onto MacConkey agar plates containing rifampicin 100 μg/mL and ampicillin 100 μg/mL, in order to count transconjugants (i.e., Lac^+^, Rif^*R*^, Amp^*R*^). Additionally, serial dilutions were plated onto MacConkey agar containing rifampicin to count recipient cells (i.e., Lac^+^, Rif^*R*^). The plates were incubated overnight at 37°C for 24 hours, and CFU counts used to obtain the frequency of transconjugants per recipient cells. The reported frequency is the average of 4 independent experiments.

### Phylogenetic Analysis

Phylogenetic analysis of 74 different ICEs from both our study and public databases^3^ was performed by SNP calling and phylogeny inference using CSI Phylogeny 1.4^4^ (Kaas et al., 2014). The entire sequences of these elements were used as inputs. All *P. mirabilis* ICEs analyzed in previous publications were included, the only exception were very similar ICEs from the same study, in which case only one representative was used. Also, representative ICEs from other species were included in the analysis, as well as previously uncharacterized elements from *P. mirabilis* found in whole genome sequences deposited in the NCBI database. Accession numbers are provided in Supplementary Table S1. The same pipeline was used to infer phylogenetic relationships of *P. mirabilis* strains containing the ICEs for which whole genome sequence is available, as well as a similar number of strains devoid of this element. Accession numbers of these genome sequences are provided in Supplementary Table S2. This analysis uses FastTree to build the approximately-maximum-likelihood trees (Price et al., 2010).

## Results

### Occurrence of SXT/R391 Elements in *P. mirabilis*

The distribution of SXT/R391 ICEs in *P. mirabilis* clinical isolates from Brazil was determined by screening a collection of isolates using a PCR strategy to detect the integrase (*int*) and *rumA* genes, two conserved core genes. Among 76 isolates, 5 carried *int* and *rumA*, indicating the presence of a type 1 SXT/R391 ICE. This represents a prevalence of 6.5% of this genetic element among these clinical isolates. All 5 SXT/R391 ICE-harboring isolates originated from urine samples. Antimicrobial resistance profiles of the ICE-harboring isolates, determined by disk diffusion method (according to CLSI guidelines) (CLSI, 2016), are shown in Table 1. Isolates PmBR19, PmBR607 and PmBR618 are multidrug-resistant (MDR) (Magiorakos et al., 2012). Nevertheless, isolates PmBR614 and PmBR595 are not MDR, despite carrying SXT/R391 ICEs.

**TABLE 1:** SXT/R391 ICEs identified in this study.

Strain	Source	ICE designation	Element Size (bp)	Max similarity with known ICEs		Resistance Profile	Resistance gene in SXT/R391	Transconjugant frequency (transconjugants/recipient)
				Most similar ICE (% coverage)	% identity within coverage			
PmBR19	Urine	ICE*Pmi*Jpn1	88,157	ICE*Pmi*Jpn1 [KY437729.1] (100)	99.99	AMC, AMP, CFL, NAL, SUT	*bla*_CMY–__2_	2.1 × 10^–8^ ± 1.3 × 10^–8^
PmBR607	Urine	ICE*Pmi*Bra607	93,418	ICE*Pmi*Jpn1 [KY437729.1] (83)	82.67	AMP, CFL, CTX, CRO, CPM, NAL, CIP, LVX, NOR, GEN, TOB, SUT	-	-
PmBR614	Urine	ICE*Pmi*Bra614	84,624	ICE*Apl*Chn1 [KX196444.1] (64)	62.5	-	-	-
PmBR595	Urine	ICE*Pmi*Bra595	75,607	ICE*Pst*33672 [CP008920.1] (99)	98.58	-	-	-
PmBR618	Urine	ICE*Pmi*Bra618	64,859	ICE*Vro*JpnAM7 [AP019798.1] (80)	77.04	AMP, CFL, CTX, CRO, CPM, NAL, CIP, GEN, TOB, SUT	-	-

*Accession numbers of ICEs from the database are given between brackets. AMC, amoxicillin-clavulanate; AMP, ampicillin; CFL, cefalotin; CIP, ciprofloxacin; CPM, cefepime; CRO, ceftriaxone; CXT, cefotaxime; GEN, gentamicin; LVX, levofloxacin; NAL, nalidixic acid; NOR, norfloxacin; SUT, sulfametoxazole/trimethoprim; TOB, tobramycin.*

### Comparative Genomic Analysis of SXT/R391 ICEs

**FIGURE 1:**
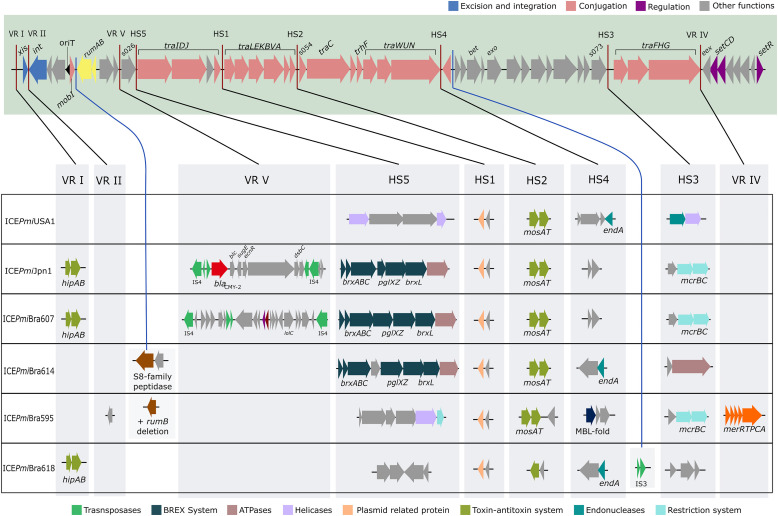
Schematic view of SXT/R391 ICEs from *P. mirabilis* from Brazil, and ICE*Pmi*USA1 (HI4320). In the shadowed box the backbone of this family of ICEs with conserved core genes is represented. Among the backbone genes, those colored blue are associated with excision and integration, genes colored pink are associated with transfer machinery, and genes colored purple are associated with regulation. Other genes with unknown function or with functions apparently not associated with transfer process are represented in gray, and the operon *rumAB* involved in error-prone DNA repair is colored yellow. Red lines indicate the position of hotspots (HS) and variable regions (VR), and genes associated with these regions in each strain in this study are represented in the scheme beneath. Blue lines represent the novel insertion points identified in this study. Similar variable structure or genes with similar putative functions in HS or VR in different ICEs were colored in the same color (as indicated below the scheme). Only the strain PmBR19 carries an antibiotic resistance gene (*bla*_CMY–__2_ in red arrow) and its ICE is identical to ICE*Pmi*Jpn1. The remainders of the ICEs were named according to the convention of the field. ICE*Pmi*Bra595 has a mercury resistance gene in VR IV (in orange color).

Isolates bearing SXT/R391 elements were subject to genome sequencing with the Illumina platform. The resulting assemblies produced the entire sequence of the ICEs contained in one single contig for isolates PmBR595, PmBR614, and PmBR618. In the assembly of Illumina data from PmBR19, the ICE was split in two different contigs. Nevertheless, we detected that its structure is identical to ICE*Pmi*Jpn1, and Sanger sequencing was used to complete the gap between the two contigs. For isolate PmBR607, the ICE was also fragmented in two or more contigs, with a structure similar to ICE*Pmi*Jpn1, but with a significant variation, since the sequence inserted in variable region V, which includes the CMY-2 beta-lactamase, was not present in any contig. To obtain the structure of this ICE, we used Nanopore sequencing. Illumina and Nanopore reads were assembled in a complete genome. A summary of the most important aspects of the ICEs, their assigned names according to the proposal of Burrus and co-workers (Burrus et al., 2006) and the characteristics of the respective bacterial strains are shown in Table 1. The structures of the ICEs are depicted in Figure 1 in comparison to the structure of the ICE*Pmi*USA1 from the type strain HI4320. The precise locations of each ICE in our genome assemblies are described in Supplementary Table S3.

BLAST analysis confirmed that the element identified in strain PmBR19 is 99.9% identical to the world-wide spread ICE*Pmi*Jpn1 (Table 1), therefore we maintained its original name. This variant carries the CMY-2 beta-lactamase gene flanked by insertion sequences in a composite transposon, inserted in variable region V (Figure 1). The only major difference between the ICE from the Brazilian strain and the one originally identified in Japan (Harada et al., 2010) is a 2478 bp deletion in the 3’-end of *pglX* gene and 456 bp deletion in the 5’-end of *pglZ* gene in the former. These genes are part of the BREX system involved in phage resistance (Goldfarb et al., 2015; Slattery et al., 2020).

Conjugation of the ICE*Pmi*Jpn1 element from *P. mirabilis* PmBr19 to *E. coli* MG1655 was achieved, albeit at a low frequency (Table 1), in the order of 2 × 10^–8^ transconjugants/recipient cell. Conjugation of the original ICE*Pmi*Jpn1 originally detected in Japan was compatible with the observed in our assays (10^–9^ transconjugants/donor) (Harada et al., 2010). Nevertheless, the same element present in strains of avian origin was transferred at much higher rates, in the order of 10^–5^ transconjugants/recipient (Aberkane et al., 2016). The conjugation frequency of other SXT/R391 ICEs vary widely (from 10^–4^ to 10^–8^), depending on the element and strain (Lei et al., 2016, 2018; Li et al., 2016; Bie et al., 2017; Siebor et al., 2018; Slattery et al., 2020). Therefore, our results are in agreement with the previously identified range of conjugation frequencies for these elements in *P. mirabilis*.

ICE*Pmi*Bra607 (PmBR607) is very similar to ICE*Pmi*Jpn1. Variable regions I, and IV, and hotspots 1-5 carry the same genes inserted in both variants (Figure 1). Nevertheless, a different composite transposon (∼10 kbp long) is located inside variable region V. This transposon is also flanked by IS*4*-like repeats, similarly to the one present in ICE*Pmi*Jpn1, but contains a number of small ORFs, none of them encoding for known resistance determinants (Figure 1). The strain bearing ICE*Pmi*Bra607 also has other noteworthy genomic features. It carries two plasmids, one of them encoding quinolone resistance (*qnrD1*), identical to plasmids previously identified among *Enterobacterales* (NZ_CP049755.1). The other plasmid (NZ_CP049754.1) has partial similarity to other enterobacterial plasmids, with a unique structure. This plasmid does not carry any resistance genes. Some pseudogenes are found in this plasmid, indicating gene decay and a lack of selective pressure for its functions in this strain, but the presence of one toxin-antitoxin pair is probably preventing its loss. Finally, the chromosome of this strain (NZ_CP049753.1) contains three integrons bearing resistance determinants, one copy of In*293* and two copies of In*2-4* (Moura et al., 2009). After the initial submission of this work, two whole genome sequences were added to the NCBI database, which contain SXT/R391 elements similar to ICE*Pmi*Jpn1 and ICE*Pmi*Bra607. We nominated these elements as ICE*Pmi*HN2p and ICE*Pmi*L901, in reference to their respective strain names (Supplementary Table S1). ICE*Pmi*L901 is highly similar to ICE*Pmi*Bra607, containing the same insertion in variable region 5. ICE*Pmi*HN2p contains both composite transposons in variable region 5: the one containing the CMY-2 beta-lactamase gene found in ICE*Pmi*Jpn1, and the one found in ICE*Pmi*Bra607, in a tandem array (data not shown).

ICE*Pmi*Bra595 (PmBR595) is highly similar to the one found in the genome of *Providencia stuartii* ATCC33672 (Frey et al., 2014). This ICE, to the best of our knowledge, does not have an official assigned designation in the literature, and has not been analyzed in depth. We hereafter refer to the *P. stuartii* element as ICE*Pst*33672 (Table 1). It contains a heavy metal resistance cassette in variable region IV (Figure 1) similar as those found in R391 and pMERPH (Böltner et al., 2002; Ryan et al., 2019), and encodes a small protein containing a metallo-beta-lactamase fold in hotspot 4, but does not carry known antimicrobial resistance determinants. Interestingly, a nonsense mutation is present in *traG* in the Brazilian isolate (data not shown), probably impairing the conjugation function of this element, given its importance to the mating-pair stabilization (Firth and Skurray, 1992).

No known antimicrobial or heavy metal resistance genes were found in ICE*Pmi*Bra614 (PmBR614) and ICE*Pmi*Bra618 (PmBR618), which have unique structures and therefore consist of new elements (Table 1, Figure 1). ICE*Pmi*Bra618 carry homologs of the *hipAB* genes in VRI, similarly to ICE*Pmi*Jpn1 and ICE*Pmi*Bra607. This toxin-antitoxin system is implicated in the formation of persisters cells (Moyed and Bertrand, 1983; Black et al., 1991; Schumacher et al., 2009), a trait that could provide selective advantage in the clinical setting for strains carrying these elements. Another toxin-antitoxin system known as *mosAT* is present in HS2 from all ICEs, except ICE*Pmi*Bra618, which carries another type of toxin without a known antitoxin. The *mosAT* (for maintenance of SXT) toxin-antitoxin system was previously shown to prevent ICE-loss from the host chromosome (Wozniak and Waldor, 2009).

### Novel Features of SXT/R391 Elements in *P. mirabilis* From Brazil

ICE*Pmi*Bra607 encodes a novel composite transposon not related to any known element in sequence databases, present in variable region V, as described above. Interestingly, one of the genes present in this novel region encodes a putative transporter of the *lolC* family, albeit the other components of this ABC transport system are not present in the transposon. Other genes encoded by this transposon may be involved in bacterial virulence, such as genes encoding for a lysozyme inhibitor (G9C79_07940) and putative adhesion proteins (G9C79_07900 and G9C79_07905). A complete list of all the genes encoded in the variable and hotspot regions of all elements is present in Supplementary Table S4.

ICE*Pmi*Bra618 has a novel insertion at HS5, not previously identified in any other ICE. This DNA sequence produces no significant hits in BLAST analysis (Supplementary Table S5). In spite of the lack of sequence conservation at the DNA level, it encodes 4 predicted proteins, which are conserved among different gammproteobacteria: two hypothetical proteins, one site-specific DNA-methyltransferase and one helix-turn-helix domain-containing protein (Supplementary Table S4).

Other interesting finding regards novel insertion points in the elements identified. VRs and HSs have been identified early in the first comparative genomic analyses of the ICEs, and later expanded to include newly observed points of DNA insertion (Beaber et al., 2002; Wozniak et al., 2009; Bie et al., 2017). In the elements identified in our study, two new points of DNA insertion have been observed. In ICE*Pmi*Bra614 and ICE*Pmi*Bra595 there is a point of DNA insertion between the 3‘ends of *mobI* and *rumB*. In ICE*Pmi*Bra618 there is a point of insertion close to HS4, where an IS*3*-like element is located (Figure 1).

Analysis of sequence similarity in DNA inserted in VRs and HSs is suggestive of a complex evolution pattern of these elements, in particular for ICE*Pmi*Bra614 and ICE*Pmi*Bra618 (Supplementary Table S5). These elements seem to be mosaics of other previously reported ICEs from several species. For instance, ICE*Pmi*Bra614 HS5 content is more similar to the insertion seen in the same position in ICE*Pmi*Chn2 (98% sequence identity), while HS4 has the same insertion as ICE*Apl*Chn1 from *Actinobacillus pleuropneumoniae* (99.9% identity). In HS3, this element contains a sequence with high similarity to ICE*Sup*CHN110003 from *Shewanella upenei* (99.9% identity). A similar convoluted pattern is seen in ICE*Pmi*Bra618, for which each insertion point is more similar to a sequence from a different ICE, from a different organism.

### Phylogenetic Relationships Between *P. mirabilis* SXT/R391 ICEs

**FIGURE 2:**
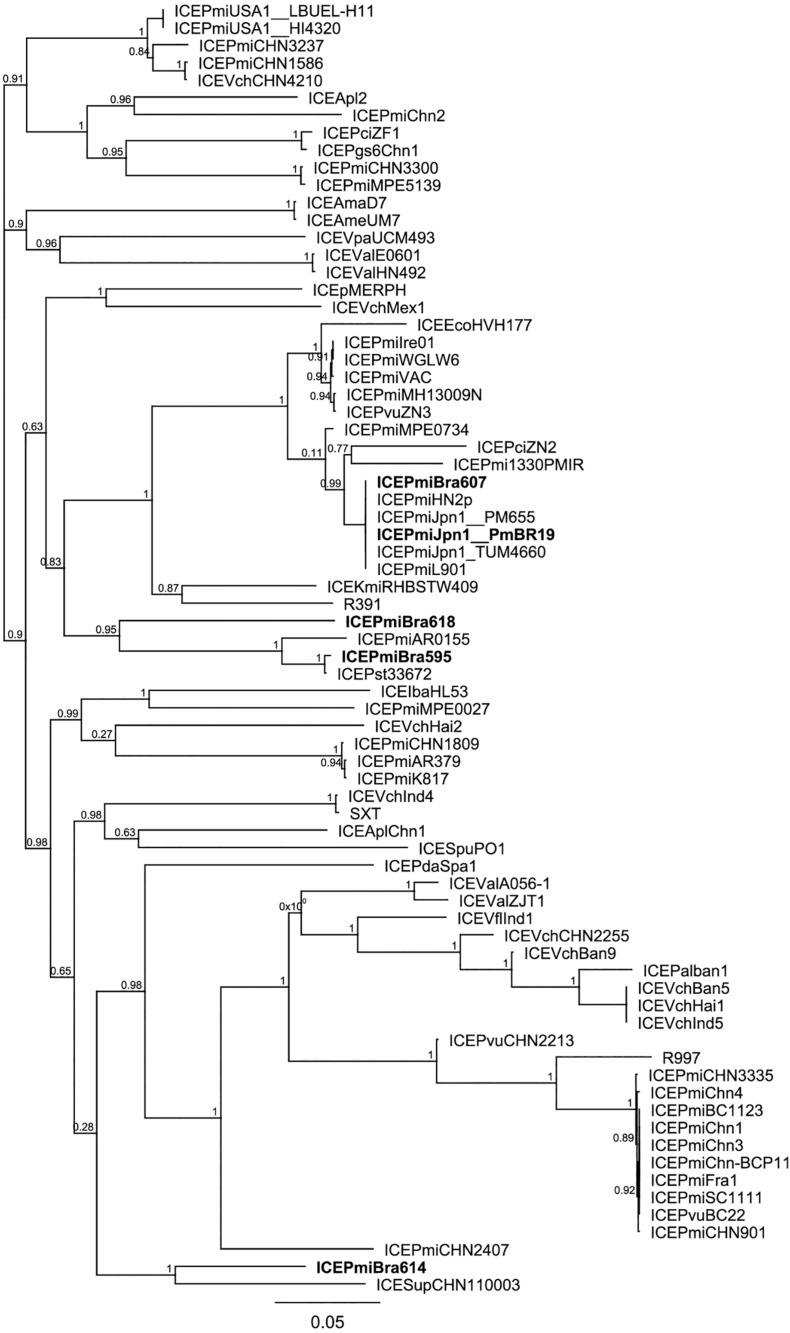
Phylogenetic relationships of SXT/R391 elements. The phylogenetic tree of SXT/R391 ICEs was built based on SNPs in their conserved regions (CSI Phylogeny pipeline) and using the approximate maximum-likelihood method in FastTree. Support values (ranging from 0 to 1) are shown next to the nodes. ICEs SXT/R391 from this study are indicated by bold letters, and strain names are shown after underscore for some ICEs found in multiple strains.

We analyzed the relationship between the ICEs identified in our study and other representative ICEs from the literature and databases. For this purpose, first we analyzed their phylogenetic relationships based on SNPs in their conserved regions using the CSI phylogeny pipeline (Kaas et al., 2014; Figure 2). As suggested by their common structure and similar content in hotspots and variable regions, ICE*Pmi*Bra607 and ICE*Pmi*Jpn1 are highly similar. ICE*Pmi*Bra595 is more similar to ICE*Pmi*Bra618, although they carry markedly different contents in variable and hotspot regions (Figure 1, Supplementary Tables S4,S5). ICE*Pmi*Bra614 is more distantly related to the other elements found in this work in *P. mirabilis* originated from Brazil. In another analysis, using a tree constructed based on the concatenated sequence of *int*, *setR*, and *setCD*, conserved genes located in both ends of the element, the close relationship of ICE*Pmi*Bra607 and ICE*Pmi*Jpn1 is confirmed. Nevertheless, the relationships among the other ICEs are changed, ICE*Pmi*Bra595 being more distantly related to the other elements from our study, and ICE*Pmi*Bra618 and ICE*Pmi*Bra614 more closely related (Supplementary Figure S1). Taken together with the seemingly mosaic nature of the ICEs described above (Supplementary Table S5), this is suggestive of recombination and shuffling of these elements.

**FIGURE 3:**
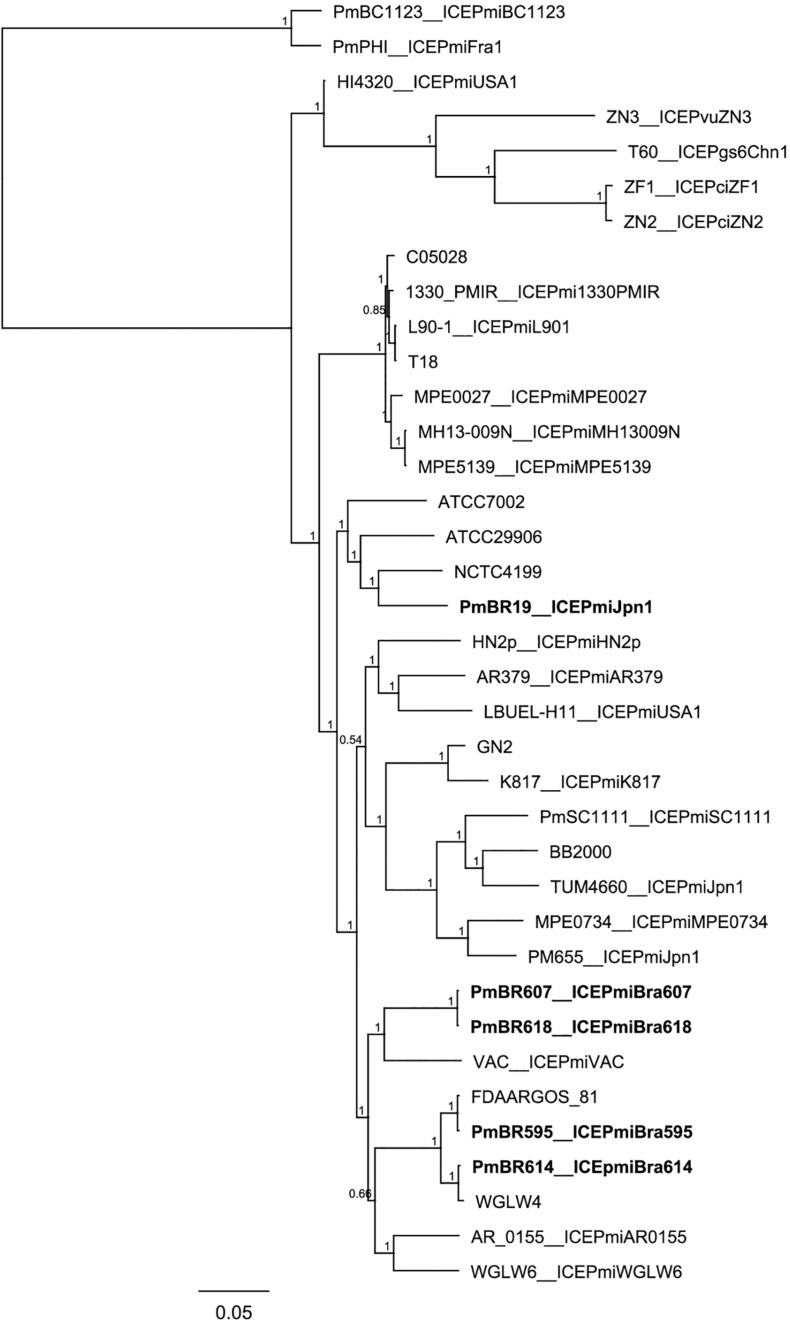
Phylogenetic tree comparing Brazilian *Proteus mirabilis* isolates with previously sequenced strains. Whole genome SNPs alignment from CSI Phylogeny analysis was used to build the tree. An approximate maximum-likelihood tree was obtained using FastTree. Brazilian strains from this study are indicated by bold letters. Strains containing the same ICEs are indicated along with corresponding ICE names after the underscore.

When compared to the species tree (Figure 3), the relationships between the ICEs show interesting features. Although clonally related and with similar resistance profiles (Figure 3, Table 1), strains PmBR607 (ICE*Pmi*Bra607) and PmBR618 (ICE*Pmi*Bra618) share ICEs with markedly different content in VRs and HSs (Figure 1, Supplementary Table S5). The Brazilian isolate PmBR19,the Japanese isolate TUM4660 and PM655 from Ireland all share the same element ICE*Pmi*Jpn1, but are not identical, suggesting horizontal transmission of this ICE among different *P. mirabilis* strains. In fact all three strains are phylogenetically closer to other *P. mirabilis* strains either devoid of SXT/R391 elements, or bearing different versions of such elements. Strain PmBR595 (bearing ICE*Pmi*Bra595) is closely related to strain FDAARGOS_81, although the later does not possess a SXT/R391 element in its genome, again indicating loss or horizontal acquisition of this element in the evolutionary history of *P. mirabilis* lineages. The same is true for strains PmBr614 and WGLW4, closely related, but the former carrying ICE*Pmi*Bra614, while the latter is devoid of any conjugative element.

### Identification of Exclusion Groups

**FIGURE 4:**
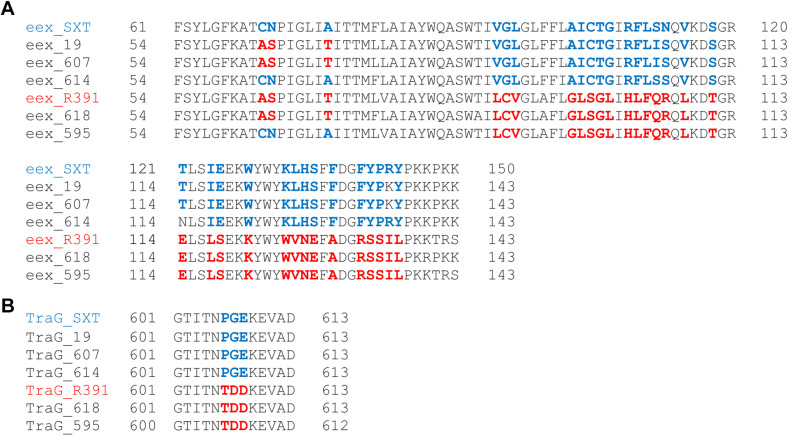
Exclusion group analysis. Comparison of the sequences of Eex **(A)** and TraG **(B)** of the five ICEs from this study to SXT and R391. The relevant variable residues are colored. In blue, residues identical to SXT and in red, residues identical to R391.

As previously described, SXT/R391 ICEs are divided in two different exclusion groups, S, related to the SXT element, and R, related to the R391 element (Marrero and Waldor, 2007). Both Eex and TraG determine the exclusion groups, which can be differentiated based on the presence of specific aminoacids in their primary sequences, in particular the 56 C-terminal aminoacids in Eex, and three specific positions in TraG. We therefore sought to determine to which exclusion group each of the five ICEs identified in this study belong to. The comparison of the relevant aminoacid residues with the ones present in SXT and R391 revealed that ICE*Pmi*Bra595 and ICE*Pmi*Bra618 belong to the R group, while ICE*Pmi*Bra614, ICE*Pmi*Bra607, and ICE*Pmi*Jpn1 belong to the S group (Figure 4).

## Discussion

SXT/R391 ICEs have been recognized in the last two decades as important agents in the dissemination of antimicrobial resistance (Burrus et al., 2006; Bioteau et al., 2018). In our work, we identified 5 of these elements in *P. mirabilis* isolated in the clinical setting in Brazil. One of these elements is the ICE*Pmi*Jpn1, which carries the CMY-2 beta-lactamase and is present in several different isolates from different origins around the globe (Harada et al., 2010; Mata et al., 2011; Aberkane et al., 2016; Lei et al., 2016; Mac Aogáin et al., 2016; Bie et al., 2017). To the best of our knowledge, this is the first description of *P. mirabilis* carrying this element in the Americas. The global spread of *P. mirabilis* strains carrying this particular variant indicates a strong selective advantage provided by this version of the ICE.

On the other hand, most of the elements identified in this work do not carry resistance determinants. Recently it has been proposed that type 1 SXT/R391 elements (i.e., those in which the ICE is inserted in the 5‘ end of *prfC*, such as the ones identified in this work) are mostly associated with the dissemination of antibiotic resistance, in the case of clinical isolates, and with heavy metal resistance, in the case of environmental isolates (Bioteau et al., 2018). This does not seem to be the case of the ICEs identified in *P. mirabilis* clinical isolates in our work, since most of them do not carry any known resistance determinants. Many of the studies searching for SXT/R391 elements were focused in multi-resistant clinical strains (Mata et al., 2011; Mac Aogáin et al., 2016; Lei et al., 2018), and this fact may have introduced a bias in the current database of known SXT/R391 elements^5^ (Liu et al., 2019). Our study aimed at the identification of such elements regardless of the antimicrobial resistance status of the strains. On the other hand, the relatively small dataset obtained in our work does not dismiss the idea that most of these elements are associated with antimicrobial resistance. It is an interesting question for further examination whether most of the type 1 elements from the SXT/R391 family are associated or not with antimicrobial resistance, and if this pattern varies in the different species in which they usually occur. More recently, another example of an element devoid of resistance genes was identified in a *P. mirabilis* isolated from wastewater (Slattery et al., 2020).

Another possibility would be that these elements carry yet uncharacterized resistance determinants. Nevertheless, for the strains characterized in this work, this is not likely to be the case. The antimicrobial resistance pattern correlates with resistance genes identified outside the ICE in the whole genome sequence of these strains, according to analysis performed on the CARD database (data not shown). These elements probably influence other aspects of cellular physiology. In one example, one SXT/R391 element from a *V. cholerae* strain has been shown to increase c-di-GMP levels in the cell, affecting motility and biofilm formation (Bordeleau et al., 2010). Given that many of the genes present in VRs and HSs encode proteins of unknown function, it is a pressing matter to understand which effects SXT/R391 elements may have in the host cell.

Genes involved in DNA repair, and toxin-antitoxin systems are other elements frequently present in SXT/R391 ICEs (Wozniak et al., 2009). These may also provide selective advantage to the host cell under certain environmental conditions. Of particular interest, many ICEs carry homologs of the *hipAB* system, involved in the formation of persisters (Moyed and Bertrand, 1983; Black et al., 1994; Schumacher et al., 2009). Interestingly, *hipAB* have been implicated in stability of R391 like *mosAT* have been demonstrated for SXT, indicating a role as addiction modules for the ICE (Wozniak and Waldor, 2009; Carraro et al., 2015). Whether these toxin-antitoxin systems could contribute to the formation of persisters is still unknown.

All ICEs from our study have the same genes inserted in HS1, suggesting a common ancestry. ICE*Pmi*Jpn1, ICE*Pmi*Bra607, and ICE*Pmi*Bra618 all have the above-mentioned *hipAB* system inserted in VRI. Nevertheless, it is interesting to note that the ICEs identified in this work share the same genes inserted in VRs and HSs with many different SXT/R391 elements. Furthermore, particularly for ICE*Pmi*Bra614 and ICE*Pmi*Bra618, DNA in each insertion point bears more similarity with a different ICE (Supplementary Table S5), which is suggestive of an intricate evolutionary path of these elements.

It is unlikely that the pattern observed in Supplementary Table S5 indicates that the same DNA sequence has entered in the same insertion point of many different ICEs in independent events in the course of evolution. A more parsimonious hypothesis would be that each DNA sequence entered at one given insertion point in one element, and these elements are being constantly shuffled by recombination. Such recombination would be possible if two different ICEs could coexist in the cell. It has already been demonstrated that there are two independent exclusion groups, which permit that two different variants to be inserted in tandem in the cell (Hochhut et al., 2001; Burrus and Waldor, 2004; Marrero and Waldor, 2007). ICE recombination has already been demonstrated experimentally, and is facilitated by the Bet/Exo recombination machinery encoded by these elements (Garriss et al., 2009, 2013). For the ICEs described in this study, we identified that ICE*Pmi*Bra618 and ICE*Pmi*Bra595 belong to the R group, and that ICE*Pmi*Jpn1, ICE*Pmi*Bra607 and ICE*Pmi*Bra614 are from the S group (Figure 4).

Our analysis also suggests many events of ICE transfer or loss among Proteae and with other bacteria. Strain PmBR595 harbors the exact same element found in *P. stuartii* (ICE*Pmi*Bra595). On the other hand, this strain is markedly genetically close to *P. mirabilis* FDAARGOS_81, which does not carry any ICE. At least one event of ICE gain or loss is clear from this observation, and the same is true for strains WGLW4 and PmBr614. Another interesting scenario is presented by strains PmBR607 (ICE*Pmi*Bra607) and PmBR618 (ICE*Pmi*Bra618). Those strains are very closely related but carry different ICEs. ICE*Pmi*Bra607 and ICE*Pmi*Bra618 carry different DNA content in VRs and HSs, and show a considerable phylogenetic distance. In this case, either multiple events of ICE gain and loss occurred in these strains, or extensive inter-ICE recombination produced new variants containing different DNA insertions in VRs and HSs.

ICE*Pmi*Jpn1 is another interesting case, since it has been detected in *P. mirabilis* isolates from many parts of the world (Mata et al., 2011; Aberkane et al., 2016; Lei et al., 2016; Mac Aogáin et al., 2016; Bie et al., 2017). In our study, we found another example of *P. mirabilis* carrying this element. Two whole genome sequences of *P. mirabilis* strains containing this element are present in the database: strains TUM4660 (the one from which this element was first isolated in Japan) and PM655 isolated in Ireland. The Brazilian strain carrying this element (PmBR19) is not closely related to either TUM4660 or PM655, according to our phylogenetic analysis. Therefore, this particular SXT/R391 variant is likely being transmitted to and maintained in different strains, which indicates a strong selective advantage, probably linked to the world-wide massive use of beta-lactams.

## Conclusion

In conclusion, our study described for the first time the genetic structure of SXT/R391 ICEs present in *P. mirabilis* in Brazil, which include three previously unknown elements. Future studies are needed to understand the prevalence of these elements, in particular the ones not carrying antimicrobial resistance genes.

## Data Availability Statement

The datasets generated for this study can be found in the NCBI database, under BioProject PRJNA576511 and genome assemblies GCA_011149675.1, GCA_009184605.1, GCA_009183735.1, GCA_009183705.1, and GCA_009183685.1.

## Ethics Statement

This study used strains obtained in the cities of São Paulo and Maringá, in Brazil. Committee for Ethics in Research (C.E.P.) from ICB, University of São Paulo, issued certificate of exemption 706/14 approving the study, which collected no patient-related information. Committee for Ethics in Research Involving Human Beings (COPEP) from State University of Maringá also approved the study (CAAE 318.0.093.000-11/COPEP UEM).

## Author Contributions

RG, JS, and MF conceived the study. JS, MF, LC, MT, MA, TS, NL, and RG conducted experimental work and data analysis. JS and RG wrote the manuscript. All authors contributed to the article and approved the submitted version.

## Conflict of Interest

The authors declare that the research was conducted in the absence of any commercial or financial relationships that could be construed as a potential conflict of interest.

## Footnotes

1 http://www.microbesng.uk

2 http://www.usadellab.org/cms/?page=trimmomatic

3 https://www.ncbi.nlm.nih.gov/genbank/

4 https://cge.cbs.dtu.dk/services/CSIPhylogeny/

5 https://db-mml.sjtu.edu.cn/ICEberg
