# Supplementary figures and images for: Genomic Analysis of SXT/R391 Integrative Conjugative Elements From Proteus mirabilis Isolated in Brazil
Source: Front Microbiol. 2020 Oct 20;11:571472. doi: 10.3389/fmicb.2020.571472 (PMC7606855; doi:10.3389/fmicb.2020.571472)

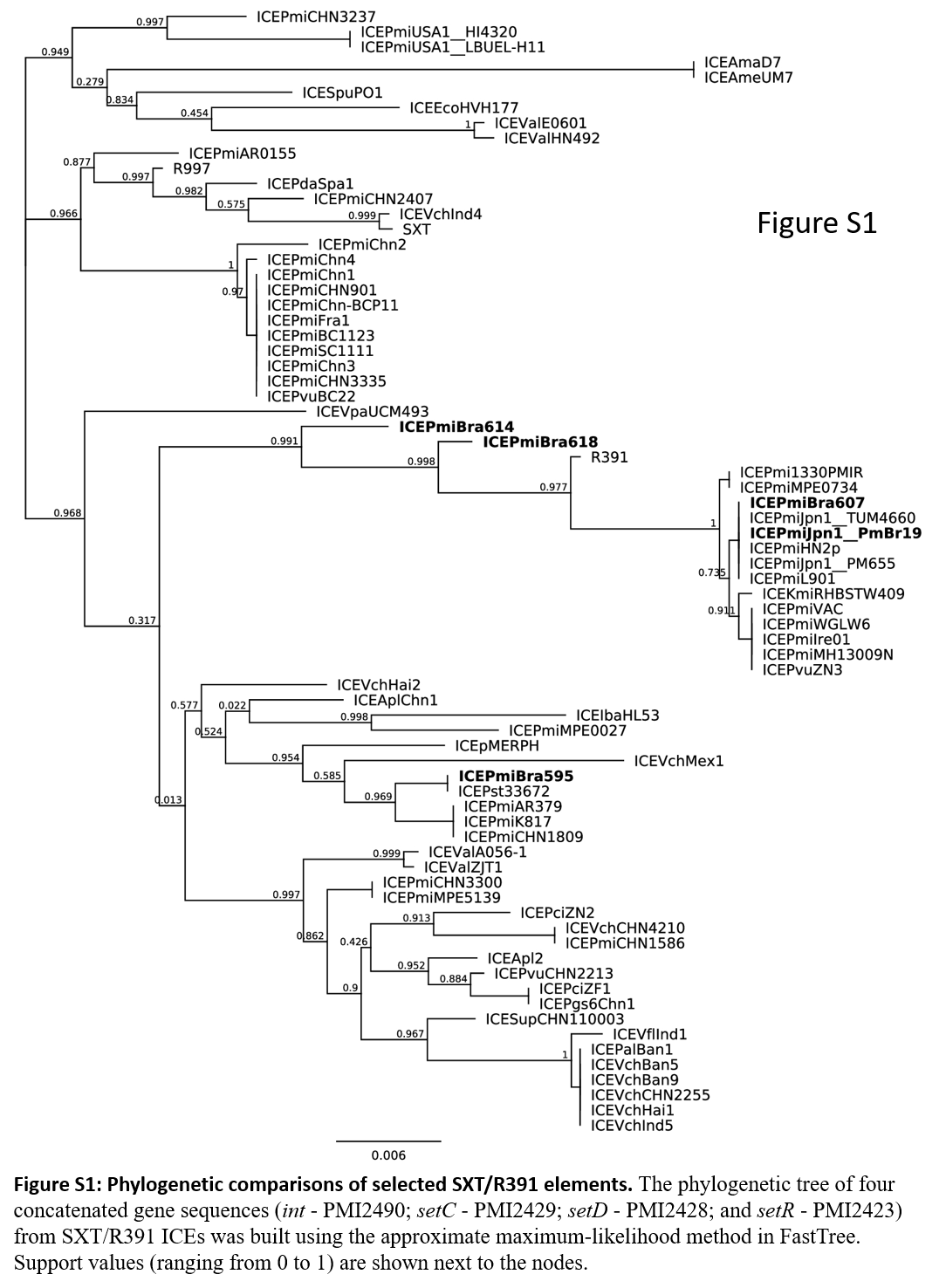

Supplement: Supplementary file 1 [file Image_1.TIF]
