# Supplementary material for: Genomic Analysis of SXT/R391 Integrative Conjugative Elements From Proteus mirabilis Isolated in Brazil
Source: Front Microbiol. 2020 Oct 20;11:571472. doi: 10.3389/fmicb.2020.571472 (PMC7606855; doi:10.3389/fmicb.2020.571472)
Supplement: Supplementary file 2 [file Table_1.docx]

**Table S1 – ICEs used in phylogenetic analysis**

| **Name** | **Strain** | **Isolation source** | **Isolation place** | **Year** | **Accession** |
| --- | --- | --- | --- | --- | --- |
| ICE*Ama*D7 | *Alteromonas macleodii* strain D7 | Seawater | Andaman Sea, Thailand | 2000 | CP014323 |
| ICE*Ame*UM7 | *Alteromonas editerrânea* UM7 | Water | Ionian sea | NA | CP004853 |
| ICE*Apl*2 | *Actinobacillus pleuropneumoniae* MIDG3553 | Pneumonic lung of a pig | UK | 2012 | MF187965 |
| ICE*Apl*Chn1 | *Actinobacillus pleuropneumoniae* app6 | Pig with respiratory disease | Shanghai, China | 2013 | KX196444 |
| ICE*Eco*HVH177 | *Escherichia coli* HVH 177 | Human blood | Denmark | 2003 | AZJM01000017 |
| ICE*Iba*HL53 | *Idiomarinaceae bacterium* HL-53 | NA | NA | NA | LN899469 |
| ICE*Kmi*RHBSTW409 | *Klebsiella michiganensis* RHBSTW-00409 | Freshwater sample | UK | 2017 | CP055325 |
| ICE*Pal*ban1 | *Providencia alcalifaciens* Ban1 | NA | Bangladesh | 1999 | GQ463139 |
| ICE*Pci*ZF1 | *Proteus cibarius* ZF1 | Swine feces | Nantong, China | 2018 | CP047340 |
| ICE*Pci*ZN2 | *Proteus cibarius* ZN2 | Swine nose swab sample | Nantong, China | 2018 | CP047349 |
| ICE*Pda*Spa1 | *Photobacterium damselae subsp. Piscicida* PC554.2 | Fish | Galicia, Spain | 2003 | AJ870986.2 |
| ICE*Pgs6*Chn1 | *Proteus genomospecies 6* (*P. columbae*) T60 | Pork | China | NA | MN507533 |
| ICEpMERPH | *Shewanella putrefaciens* pMERPH | River mersey | UK | 1987 | MH974755 |
| ICE*Pmi*1330PMIR | *Proteus mirabilis* 1330_PMIR | Wound | USA | 2013 | JVTJ00000000 |
| ICE*Pmi*AR0155 | *Proteus mirabilis* AR_0155 | NA | NA | NA | CP021694 |
| ICE*Pmi*AR379 | *Proteus mirabilis* AR379 | NA | NA | NA | CP029133 |
| ICE*Pmi*BC1123 | *Proteus mirabilis* PmBC1123 | Swine | Mianyang, China | 2017 | CP034091 |
| ICE*Pmi*Chn1 | *Proteus mirabilis* PM13C04 | Chicken fecal sample | Hubei, China | 2013 | KT962845 |
| ICE*Pmi*Chn2 | *Proteus mirabilis* JN7 | Broiler carcasses | Shandong, China | 2013 | KY437726 |
| ICE*Pmi*Chn3 | *Proteus mirabilis* JN28 | Broiler carcasses | Shandong, China | 2013 | KY437727 |
| ICE*Pmi*Chn4 | *Proteus mirabilis* JN49 | Broiler carcasses | Shandong, China | 2013 | KY437728 |
| ICE*Pm*iCHN901 | *Proteus mirabilis* MD20140901 | Stool | Beijing, China | 2014 | KX243408 |
| ICE*Pmi*CHN1586 | *Proteus mirabilis* 08MAS1586 | Food | Maanshan, China | 2008 | KX243404 |
| ICE*Pmi*CHN1809 | *Proteus mirabilis T*J1809 | Stool | Tianjin, China | 2013 | KX243413 |
| ICE*Pmi*CHN2407 | *Proteus mirabilis* 09MAS2407 | Stool | Maanshan, China | 2008 | KX243405 |
| ICE*Pmi*CHN3237 | *Proteus mirabilis* TJ3237 | Stool | Tianjin, China | 2013 | KX243414 |
| ICE*Pmi*CHN3300 | *Proteus mirabilis* TJ3300 | Stool | Tianjin, China | 2013 | KX243415 |
| ICE*Pmi*CHN3335 | *Proteus mirabilis* TJ3335 | Stool | Tianjin, China | 2013 | KX243416 |
| ICE*Pmi*Chn-BCP11 | *Proteus mirabilis* BCP11 | Pig fecal swab | Sichuan, China | 2016 | MG773277 |
| ICE*Pmi*Fra1 | *Proteus mirabilis* PmPHI | Stool | France | 2012 | MF490434 |
| ICE*Pmi*HN2p | *Proteus mirabilis* HN2p | Swine | Henan, China | 2019 | CP046048 |
| ICE*Pmi*Ire01 | *Proteus mirabilis* Ire01 | Wastewater | Dublin, Ireland | 2018 | MN520463 |
| ICE*Pmi*Jpn1 | *Proteus mirabilis* PM655 | Urine | Dublin, Ireland | 2013 | JSUO01000115 |
| ICE*Pmi*Jpn1 | *Proteus mirabilis* TUM4660 | Soft tissue swab | Japan | 2008 | BGMB00000000 |
| ICE*Pmi*K817 | *Proteus mirabilis* K817 | NA | Kielce, Poland | 2002 | CP044028 |
| ICE*Pmi*L901 | *Proteus mirabilis* L90-1 | Stool | Hangzhou, China | 2016 | CP045257 |
| ICE*Pmi*MH13009N | *Proteus mirabilis* MH13-009N | NA | Hanoi, Viet Nam | 2013 | BFCK00000000 |
| ICE*Pmi*MPE0027 | *Proteus mirabilis* MPE0027 | Feces | Shanghai, China | 2018 | CP053683 |
| ICE*Pmi*MPE0734 | *Proteus mirabilis* MPE0734 | Malayan Pangolin | Shenzhen, China | 2018 | CP053615 |
| ICE*Pmi*MPE5139 | *Proteus mirabilis* MPE5139 | Feces | Guangzhou, China | 2019 | CP053684 |
| ICE*Pmi*SC1111 | *Proteus mirabilis* PmSC1111 | Swine | Mianyang, China | 2017 | CP034090 |
| ICE*Pmi*USA1 | *Proteus mirabilis* HI4320 | Human urine | Maryland, USA | 1986 | AM942759 |
| ICE*Pmi*USA1 | *Proteus mirabilis* LBUEL-H11 | Tracheal secretion | Londrina, Brazil | 2015 | QGGA01000000 |
| ICE*Pmi*VAC | *Proteus mirabilis* VAC | Rectal screening | France | 2016 | CP042907 |
| ICE*Pmi*WGLW6 | *Proteus mirabilis* WGLW6 | NA | NA | NA | AMGT00000000 |
| ICE*Pst*33672 | *Providencia stuartii* ATCC 33672 | NA | NA | NA | CP008920 |
| ICEPvuChnBC22 | *Proteus vulgaris* BC22 | Swine anal swab | China | 2018 | MH160822 |
| ICE*Pvu*CHN2213 | *Proteus vulgaris* 08MAS2213 | Food | Maansham, China | 2008 | KX243403 |
| ICE*Pvu*ZN3 | *Proteus vulgaris* ZN3 | Swine nose swab | Nantong, China | 2018 | CP047344 |
| ICE*Spu*PO1 | *Shewanella putrefaciens* W3-18-1 | Marine sediments | Pacific Ocean | 2000 | CP000503 |
| ICE*Sup*CHN110003 | *Shewanella upenei* 110003 | Stool | Dangtu, China | 2011 | MG014393 |
| ICE*Val*A056-1 | *Vibrio alginolyticus A*056 | Whiteleg shrimp | Guangdong, China | 2003 | KR231688 |
| ICE*Val*E0601 | *Vibrio alginolyticus* E0601 | Seawater | Guangdong, China | 2006 | KT072768 |
| ICE*Val*HN492 | *Vibrio alginolyticus* HN492 | Seawater | Guangdong, China | 2008 | KT072769 |
| ICE*Val*ZJT1 | *Vibrio alginolyticus* ZJ-T | Orange-spotted grouper | Guangdong, China | 2005 | CP016224 |
| ICE*Vch*Ban5 | *Vibrio cholerae* Ban5 | NA | Bangladesh | 1998 | GQ463140 |
| ICE*Vch*Ban9 | *Vibrio cholerae* MJ-1236 | NA | Bangladesh | 1994 | CP001485 |
| ICE*Vch*CHN2255 | *Vibrio cholerae* ICDC-2255 | Patient | Hainan, China | 2008 | KT151660 |
| ICE*Vch*CHN4210 | *Vibrio cholerae* ICDC-4210 | Patient | Jiangxi, China | 1999 | KT151662 |
| ICE*Vch*Hai1 | *Vibrio cholerae* VC1786 | Stool | Artibonite, Haiti | 2010 | JN648379 |
| ICE*Vch*Hai2 | *Vibrio cholerae* HC-1A2 | Stool | Haiti | 2010 | AJRO00000000 |
| ICE*Vch*Ind4 | *Vibrio cholerae* Ind4 | Clinical | Kolkata, India | 1997 | GQ463141 |
| ICE*Vch*Ind5 | *Vibrio cholerae* Ind5 | NA | Sevagram, India | 1994 | GQ463142 |
| ICE*Vch*Mex1 | *Vibrio cholerae* Mex1 | Sewage | San Luis Potosi, Mexico | 2001 | GQ463143 |
| ICE*Vfl*Ind1 | *Vibrio fluvialis* H08942 | Diarrhea | Kolkata, India | 2002 | KM213605 |
| ICE*Vpa*UCM493 | *Vibrio parahaemolyticus* UCM-V493 | Sediment | Spain | 2002 | CP007004 |
| R391 | *Providencia rettgeri 107* | NA | Pretoria, South Africa | 1967 | AY090559 |
| R997 | *Proteus mirabilis* R997 | NA | India | 1977 | KY433363 |
| SXT | *Vibrio cholerae* MO10 | Clinical | Chennai, India | 1992 | AY055428 |

**NA: information not available**
