# Supplementary material for: Genomic Analysis of SXT/R391 Integrative Conjugative Elements From Proteus mirabilis Isolated in Brazil
Source: Front Microbiol. 2020 Oct 20;11:571472. doi: 10.3389/fmicb.2020.571472 (PMC7606855; doi:10.3389/fmicb.2020.571472)
Supplement: Supplementary file 3 [file Table_2.DOCX]

**Table S2 – *P. mirabilis* strains used in phylogenetic analysis**

| **Strain** | **ICE SXT/R391** | **Source** | **Isolation Place** | **Year** | **Accession** |
| --- | --- | --- | --- | --- | --- |
| 1330_PMIR | ICEPmi1330PMIR | Wound | USA | 2013 | JVTJ00000000 |
| AR379 | ICEPmiAR379 | NA | NA | NA | CP029133 |
| AR_0155 | ICEPmiAR0155 | NA | NA | NA | CP021694 |
| ATCC 7002 | No | NA | NA | NA | JOVJ00000000 |
| ATCC 29906 | No | NA | NA | NA | ACLE00000000 |
| BB2000 | No | NA | Maryland, USA | NA | NC_022000 |
| BC11-24 | No | Pig liver | Sichuan, China | 2016 | CP026571 |
| C05028 | No | Stool | Guangdong, China | 2005 | ANBT00000000 |
| FDAARGOS_81 | No | Urine | USA | 2013 | CP026062 |
| GN2 | No | Urine | Beijing, China | 2014 | CP026581 |
| HI4320 | ICEPmiUSA1 | Human Urine | Maryland, USA | 1986 | AM942759 |
| HN2p | ICEPmiHN2p | Swine | Henan, China | 2019 | CP046048 |
| K670 | No | NA | Kielce, Poland | 2000 | CP028356 |
| K817 | ICEPmiK817 | NA | Kielce, Poland | 2002 | CP044028 |
| L90-1 | ICEPmiL901 | Stool | Hangzhou, China | 2016 | CP045257 |
| LBUEL-H11 | ICEPmiUSA1 | Tracheal secretion | Londrina, Brazil | 2015 | QGGA01000000 |
| MH13-009N | ICEPmiMH13009N | NA | Hanoi, Viet Nam | 2013 | BFCK01000000 |
| MPE0027 | ICEPmiMPE0027 | Feces | Shanghai, China | 2018 | CP053683 |
| MPE0734 | ICEPmiMPE0734 | Malayan Pangolin | Shenzen, China | 2018 | CP053615 |
| MPE5139 | ICEPmiMPE5139 | Feces | Guangzhou, China | 2019 | CP053684 |
| NCTC4199 | No | Stool | London, UK | 1933 | LR134205 |
| PM655 | ICE*Pmi*Jpn1 | Urine | Dublin, Ireland | 2013 | JSUO00000000 |
| PmBC1123 | ICEPmiBC1123 | Swine | Mianyang, China | 2017 | CP034091 |
| PmPHI | ICEPmiFra1 | Stool | France | 2012 | PUXR00000000 |
| PmSC1111 | ICEPmiSC1111 | Swine | Mianyang, China | 2017 | CP034090 |
| T18 | No | Urine | Zhejiang, China | 2014 | CP017085 |
| T60 (*P. columbae*) | ICE*Pgs6Chn1* | Pork | China | NA | CP043925 |
| TUM4660 | ICEPmiJpn1 | Soft tissue swab | Japan | 2008 | BGMB00000000 |
| VAC | ICEPmiVAC | Rectal screening | France | 2016 | CP042907 |
| WGLW4 | No | NA | NA | NA | AMGU00000000 |
| WGLW6 | ICEPmiWGLW6 | NA | NA | NA | AMGT00000000 |
| ZA25 | No | Swine | Nantong, China | 2018 | CP047352 |
| ZF1 (*P.cibarius*) | ICEPciZF1 | Swine feces | Nantong, China | 2018 | CP047340 |
| ZN2 (*P.cibarius*) | ICEPciZN2 | Swine nose swab | Nantong, China | 2018 | CP047349 |
| ZN3 (*P.vulgaris*) | ICEPvuZN3 | Swine nose swab | Nantong, China | 2018 | CP047344 |

**No: SXT/R391 element not detected in the genome. NA: no information is available.**
