# Supplementary material for: Genomic Analysis of SXT/R391 Integrative Conjugative Elements From Proteus mirabilis Isolated in Brazil
Source: Front Microbiol. 2020 Oct 20;11:571472. doi: 10.3389/fmicb.2020.571472 (PMC7606855; doi:10.3389/fmicb.2020.571472)
Supplement: Supplementary file 4 [file Table_3.DOCX]

**Table S3 – Accession information of the ICEs identified in this study**

| **Element** | **Contig or chromosome accession number** | **ICE coordinates^a^** |
| --- | --- | --- |
| ICE*Pmi*Jpn1 | WEKK01000002.1 | 311319...223108 |
| ICE*Pmi*Bra595 | WEKH01000004.1 | 55053...130714 |
| ICE*Pmi*Bra607 | CP049753.1 | 1687350...1780822 |
| ICE*Pmi*Bra614 | WEKJ01000004.1 | 54801...139479 |
| ICE*Pmi*Bra618 | WEKI01000001.1 | 130187...195100 |

^a^ Coordinates comprise the entire sequence of the ICE, including the repeats *attL* and *attR*.
