# Supplementary material for: Genomic Analysis of SXT/R391 Integrative Conjugative Elements From Proteus mirabilis Isolated in Brazil
Source: Front Microbiol. 2020 Oct 20;11:571472. doi: 10.3389/fmicb.2020.571472 (PMC7606855; doi:10.3389/fmicb.2020.571472)
Supplement: Supplementary file 5 [file Table_4.DOCX]

Table S4 – Gene content in hotspots and variable regions

| **ICE** | **HS/VR** | **Locus** | **Anotation** |
| --- | --- | --- | --- |
| ICE*Pmi*Jpn1 | HS1 | GBN10_02810 | Plasmid-related protein |
|  |  | GBN10_02805 | hypothetical protein |
|  | HS2 | GBN10_02770 | MosA |
|  |  | GBN10_02765 | MosT |
|  | HS3 | GBN10_02650 | hypothetical protein |
|  |  | GBN10_02645 | McrB (AAA domain-containing protein) |
|  |  | GBN10_02640 | McrC (restriction endonuclease) |
|  | HS4 | GBN10_02725 | hypothetical protein |
|  |  | GBN10_02720 | hypothetical protein |
|  | HS5 | GBN10_02865 | BrexA (DUF1819 family protein) |
|  |  | GBN10_02860 | BrexB (DUF1788 domain-containing protein) |
|  |  | GBN10_02855 | BrexC |
|  |  | GBN10_02850 | PglX |
|  |  | GBN10_02845 | PglZ |
|  |  | GBN10_02840 | BrxL |
|  |  | GBN10_02835 | AAA family ATPase |
|  | VR I | GBN10_02990 | HipB (helix-turn-helix domain-containing protein) |
|  |  | GBN10_02985 | type II toxin-antitoxin system HipA family toxin |
|  | VR V | GBN10_02940 | IS4-like element ISVsa5 family transposase |
|  |  | GBN10_02935 | IS1380 family transposase |
|  |  | GBN10_02930 | IS1380 family transposase |
|  |  | GBN10_02925 | class C beta-lactamase CMY-2 |
|  |  | GBN10_02920 | blc |
|  |  | GBN10_02915 | quaternary ammonium compound-resistance protein SugE |
|  |  | GBN10_02910 | LuxR family transcriptional regulator |
|  |  | GBN10_02905 | DUF4165 domain-containing protein |
|  |  | GBN10_02900 | thioredoxin fold domain-containing protein |
|  |  | GBN10_02895 | TraC family protein |
|  |  | GBN10_02890 | IS1380-like element ISEc9 family transposase |
|  |  | GBN10_02885 | IS4-like element ISVsa5 family transposase |
|  |  | GBN10_02880 | lipopolysaccharide core heptose(II) kinase RfaY |
|  |  | GBN10_02875 | Hypothetical protein |
| ICE*Pmi*Bra607 | HS1 | G9C79_08035 | Plasmid-related protein |
|  |  | G9C79_08040 | hypothetical protein |
|  | HS2 | G9C79_08075 | MosA |
|  |  | G9C79_08080 | MosT |
|  | HS3 | G9C79_08200 | hypothetical protein |
|  |  | G9C79_08205 | McrB (AAA domain-containing protein) |
|  |  | G9C79_08210 | McrC (restriction endonuclease) |
|  | HS4 | G9C79_08120 | hypothetical protein |
|  |  | G9C79_08125 | hypothetical protein |
|  | HS5 | G9C79_07980 | BrexA (DUF1819 family protein) |
|  |  | G9C79_07985 | BrexB (DUF1788 domain-containing protein) |
|  |  | G9C79_07990 | BrexC |
|  |  | G9C79_07995 | PglX |
|  |  | G9C79_08000 | PglZ |
|  |  | G9C79_08005 | BrxL |
|  |  | G9C79_08010 | AAA family ATPase |
|  | VR I | G9C79_07795 | HipB (helix-turn-helix domain-containing protein) |
|  |  | G9C79_07800 | type II toxin-antitoxin system HipA family toxin |
|  | **VR V** | **G9C79_07855** | **IS4-like element ISVsa5 family transposase** |
|  |  | **G9C79_07860** | **hypothetical protein** |
|  |  | **G9C79_07865** | **CcgAII protein** |
|  |  | **G9C79_07870** | **hypothetical protein** |
|  |  | **G9C79_07875** | **ClbS/DfsB family four-helix bundle protein** |
|  |  | **G9C79_07880** | **ParA family protein** |
|  |  | **G9C79_07885** | **hypothetical protein** |
|  |  | **G9C79_07890** | **Transposase** |
|  |  | **G9C79_07895** | **IS200/IS605 family transposase** |
|  |  | **G9C79_07900** | **hypothetical protein** |
|  |  | **G9C79_07905** | **Virulence protein** |
|  |  | **G9C79_07910** | **DUF5397 family protein** |
|  |  | **G9C79_07915** | **XRE family transcriptional regulator** |
|  |  | **G9C79_07920** | **type II toxin-antitoxin system RelE/ParE family toxin** |
|  |  | **G9C79_07925** | **DUF883 family protein** |
|  |  | **G9C79_07930** | **hypothetical protein** |
|  |  | **G9C79_07935** | **hypothetical protein** |
|  |  | **G9C79_07940** | **inhibitor of g-type lysozyme** |
|  |  | **G9C79_07945** | **LolC** |
|  |  | **G9C79_07950** | **hypothetical protein** |
|  |  | **G9C79_07955** | **hypothetical protein** |
|  |  | **G9C79_07960** | **hypothetical protein** |
|  |  | **G9C79_07965** | **omptin family outer membrane protease** |
|  |  | **G9C79_07970** | **IS4-like element ISVsa5 family transposase** |
| ICE*Pmi*Bra614 | HS1 | GBN13_03985 | Plasmid-related protein |
|  |  | GBN13_03990 | hypothetical protein |
|  | HS2 | GBN13_03985 | MosA |
|  |  | GBN13_04030 | MosT |
|  | HS3 | GBN13_04145 | hypothetical protein |
|  |  | GBN13_04150 | AAA family ATPase |
|  | HS4 | GBN13_04070 | DUF4365 domain-containing protein |
|  |  | GBN13_04075 | deoxyribonuclease I |
|  | HS5 | GBN13_03925 | BrexA (DUF1819 family protein) |
|  |  | GBN13_03930 | BrexB (DUF1788 domain-containing protein) |
|  |  | GBN13_03935 | BrexC |
|  |  | GBN13_03940 | DUF262 domain-containing protein |
|  |  | GBN13_03945 | PglX |
|  |  | GBN13_03950 | PglZ |
|  |  | GBN13_03955 | BrxL |
|  |  | GBN13_03960 | AAA family ATPase |
|  | **New insertion 1** | **GBN13_03890** | **S8 family serine peptidase** |
|  |  | **GBN13_03895** | **AAA family ATPase** |
| ICE*Pmi*Bra595 | HS1 | GBN12_03285 | Plasmid-related protein |
|  |  | GBN12_03290 | hypothetical protein |
|  | HS2 | GBN12_03325 | MosA |
|  |  | GBN12_03330 | MosT |
|  |  | GBN12_03335 | hypothetical protein |
|  | HS3 | GBN12_03455 | hypothetical protein |
|  |  | GBN12_03460 | McrB (AAA domain-containing protein) |
|  |  | GBN12_03465 | McrC (restriction endonuclease) |
|  | HS4 | GBN12_03375 | MBL fold metallo-hydrolase |
|  |  | GBN12_03380 | ester cyclase |
|  |  | GBN12_03385 | helix-turn-helix domain-containing protein |
|  | HS5 | GBN12_03235 | ribbon-helix-helix protein, CopG family |
|  |  | GBN12_03240 | DUF499 domain-containing protein |
|  |  | GBN12_03245 | DUF3780 domain-containing protein |
|  |  | GBN12_03250 | DUF1156 domain-containing protein |
|  |  | GBN12_03255 | DEAD/DEAH box helicase |
|  |  | GBN12_03260 | restriction endonuclease |
|  | VR II | GBN12_03185 | DUF3892 domain-containing protein |
|  | VR IV | GBN12_03485 | MerR |
|  |  | GBN12_03490 | MerT (mercury transporter) |
|  |  | GBN12_03495 | MerP |
|  |  | GBN12_03500 | MerC |
|  |  | GBN12_03505 | MerA (mercury(II) reductase) |
|  | **New insertion 1** | **GBN12_03210** | **S8 family serine peptidase** |
| ICE*Pmi*Bra618 | HS1 | GBN11_00670 | Plasmid-related protein |
|  |  | GBN11_00675 | hypothetical protein |
|  | HS2 | GBN11_00710 | nucleotidyl transferase AbiEii/AbiGii toxin family protein |
|  |  | GBN11_00715 | hypothetical protein |
|  | HS3 | GBN11_00835 | hypothetical protein |
|  |  | GBN11_00840 | hypothetical protein |
|  |  | GBN11_00845 | hypothetical protein |
|  | HS4 | GBN11_00755 | DUF4365 domain-containing protein |
|  |  | GBN11_00760 | deoxyribonuclease I |
|  | **HS5** | **GBN11_00630** | **hypothetical protein** |
|  |  | **GBN11_00635** | **SAM-dependent methyltransferase** |
|  |  | **GBN11_00640** | **hypothetical protein** |
|  |  | **GBN11_00645** | **helix-turn-helix domain-containing protein** |
|  | VR I | GBN11_00570 | HipB (helix-turn-helix domain-containing protein) |
|  |  | GBN11_00575 | type II toxin-antitoxin system HipA family toxin |
|  | **New insertion 2** | **GBN11_00770** | **IS3 family transposase** |

Regions uniquely observed in ICEs from our study are shown in bold.
