# Supplementary material for: Genomic Analysis of SXT/R391 Integrative Conjugative Elements From Proteus mirabilis Isolated in Brazil
Source: Front Microbiol. 2020 Oct 20;11:571472. doi: 10.3389/fmicb.2020.571472 (PMC7606855; doi:10.3389/fmicb.2020.571472)
Supplement: Supplementary file 6 [file Table_5.DOCX]

**Table S5. BLAST analysis of DNA inserted in VRs and HSs in SXT/R391 elements identified in this work.**

| **SXT/R391** | **VRI** | **VRV** | **HS5** | **HS2** | **HS4** | **HS3** | **VRIV** |
| --- | --- | --- | --- | --- | --- | --- | --- |
| **ICE*Pmi*Bra607** | ICE*Pmi*HN2p (100/100)  ICE*Pmi*MPE0734 (100/100)  ICE*Pmi*L90-1 (100/100)  ICE*Pvu*ZN3 (100/100) | ICE*Pmi*HN2p (100/99.7)  ICE*Pmi*L90-1 (100/99.9) | ICE*Pmi*Ire01 (92/98)  ICE*Pvu*ZN3 (92/98)  ICE*Pmi*VAC (92/98)  ICE*Pmi*HN2p (94/98) | ICE*Pmi*HN2p (100/99.9)  ICE*Pmi*MPE0734 (100/99.9)  ICE*Pmi*L90-1 (100/99.9)  ICE*Pmi*Jpn1* (100/99.9) | ICE*Pmi*HN2p (100/100)  ICE*Pmi*L90-1 (100/100)  ICE*Pmi*Jpn1* (100/100)  ICEVflInd1 (100/100) | ICE*Pmi*HN2p (100/100)  ICE*Pmi*MPE0734 (100/100)  ICE*Pmi*L90-1 (100/100)  ICE*Pmi*Jpn1 (100/100) | - |
| **ICE*Pmi*Bra614** | - | - | ICE*Pmi*MPE5139 (91/98)  ICE*Pmi*Chn2 (91/98)  ICE*Pmi*CHN3300 (91/98)  ICE*Vch*Ban9 (91/97.9) | ICE*Apl*Chn1 (100/98.7)  ICE*Pmi*Ire01 (100/98.5)  ICE*Pvu*ZN3 (100/98.5)  ICE*Pmi*VAC (100/98.5) | ICE*Apl*Chn1 (100/99.9) | ICE*Sup*CHN110003 (100/99.9)  *ICEPmiChn2* (100/97.3) | - |
| **ICE*Pmi*Bra595** | - | - | ICE*Pst*33672 (100/99.9)  ICE*Pmi*K817 (90/97.8)  ICE*Pmi*AR379 (90/97.8)  ICE*Pmi*CHN1809 (90/97.8) | ICE*Pst*33672 (100/100)  ICE*Pmi*AR0155 (100/99.9)  ICE*Vch*MZ03 (100/98.2)  ICE*Vch*W10G (100/98.1) | ICE*Pst*33672 (100/100)  ICE*Pmi*AR0155 (100/99.9)  ICE*Pmi*BC1123 (100/92.9) | ICE*Pst*33672 (100/100)  ICE*Pmi*AR0155 (100/99.9)  ICE*Pmi*Ire01 (100/99.9)  ICE*Pvu*ZN3 (100/99.9) | ICE*Pst*33672 (100/100)  ICE *Providencia sp.* WCHPHu000369 (100/99.9)  ICE*Pmi*CHN2407 (100/99.9)  ICEpMERPH (100/99.9) |
| **ICE*Pmi*Bra618** | ICE*Pmi*Jpn1 (100/99.7)  ICE*Pmi*HN2p (100/99.7)  ICE*Pmi*MPE0734 (100/99.7)  ICE*Pmi*L90-1 (100/99.7) | - | **Encodes 4 proteins not previously related to ICEs. No significant similarity at the DNA level** | ICE*Vsp*Por1 (100/99)  ICE*Vsc*Spa3 (100/99)  ICE*Vsp*Spa3 (100/99)  *Pseudoalteromonas sp.* DL-6 (100/98.5) | ICE*Apl*Chn1 (100/99.5) | ICE *Providencia sp.* WCHPHu000369 (100/99.7) | - |

The best four BLAST hits covering at least 90% of the sequence and showing similarity ≥ 90% are shown for each HS or VR DNA sequence. Coverage/identity is shown between brackets. Each cell is colored according to the best BLAST hit, so that sequences from the same genome are in the same color.

* ICEPmiJpn1 present in three different strains.
